# The role of the public health service in the implementation of heat health action plans for climate change adaptation in Germany: A qualitative study

**DOI:** 10.1186/s12961-024-01231-6

**Published:** 2024-12-05

**Authors:** Karin Geffert, Stephan Voss, Eva Rehfuess, Bernd Rechel

**Affiliations:** 1 Chair of Public Health and Health Services Research, Institute of Medical Information Processing, Biometry and Epidemiology (IBE), Faculty of Medicine, LMU Munich, Munich, Germany; 2 Pettenkofer School of Public Health, Munich, Germany; 3 London School of Hygiene and Tropical Medicine, European Observatory On Health Systems and Policies, London, UK

**Keywords:** Heat health action plan, Public health service, Climate change, Hot temperature

## Abstract

**Background:**

In response to climate change-induced increases in heat periods, the WHO recommends the implementation of heat health action plans (HHAPs). In Germany, HHAPs are implemented neither comprehensively nor nationwide. Several recommendations have identified the public health service (PHS) at municipal and federal state levels as a key actor regarding to heat and health. Therefore, this study aimed at assessing the role of the PHS in implementing HHAPs at municipal and federal state levels in Germany.

**Methods:**

We conducted a policy document analysis to assess the legal basis for the work of the PHS in the 16 federal states in Germany. Furthermore, we conducted semi-structured interviews with 16 experts from within and outside the PHS to explore their perceptions of the PHS in the implementation of HHAPs. The interviews were analysed using reflective thematic analysis.

**Results:**

The policy document analysis revealed that heat is not mentioned in any of the federal states’ regulatory frameworks for the PHS, while tasks related to environment and health are addressed, but tend to remain vague. The interviews confirmed that there is currently no clearly defined role for the PHS in implementing HHAPs in Germany and that the actual role primarily depends on the local setting. Main barriers and facilitators could be assigned to three levels (individual, organizational and political), and two overarching contextual factors (awareness of the need for adaptation and existence of other public health emergencies) influenced the implementation of HHAPs across all levels. At the individual level, motivation, knowledge and competencies, and previous experience were possible barriers or enablers. At the organizational level, administrative structures, financial and human resources, leadership and networks were barriers or facilitators, while at the political level they included legislation and political decisions.

**Conclusions:**

The PHS could and should be a relevant actor for implementing measures addressing health and climate change locally, in particular because of its focus on vulnerable populations. However, our findings suggest that the legal basis in the federal states of Germany is insufficient. Tailored approaches are needed to overcome barriers such as rigid, non-agile administrative structures and competing priorities, while taking advantage of facilitators such as awareness of relevant actors.

**Supplementary Information:**

The online version contains supplementary material available at 10.1186/s12961-024-01231-6.

## Background

Climate change represents the greatest threat to public health in the 21st century [1]. Climate change will impact health in many different ways, including through the rise in global temperature, leading to prolonged heat periods in various parts of the world, including Germany [2]. These prolonged heat periods have various detrimental effects on human health, ranging from headaches and other mild symptoms to life-threatening conditions such as heat shock. Therefore, heat waves are associated with an increase in morbidity and mortality, but also with increases in emergency consultations and hospitalizations [3]. Estimates of annual premature heat deaths in Germany range from 1000 (year 2021) to more than 6000 (year 2019) [4].

To deal with this challenge, the WHO recommends the implementation of heat health action plans (HHAPs), with the aim of harmonizing intersectoral approaches to prevent heat-related adverse health effects by, among others, communicating warnings on the health impact of extreme heat events to key groups and settings. Vulnerable populations, such as elderly people, people with chronic conditions or people of a lower socio-economic status, are at higher risk of heat-related illness and should be the focus of such interventions [5]. In Germany, a growing number of communities and federal states have started to implement elements of HHAPs. However, HHAPs are implemented neither comprehensively nor nationwide [6]. Awareness of the topic is increasing, including in political debates in the 16 federal state parliaments, but actual implementation of HHAPs remains limited [7]. A previous study identified bottlenecks at the municipal level for the implementation of HHAPs, including lack of finances, resources, political will and knowledge, but also enablers, such as personal motivation and awareness [8]. This was also shown in a previous study that investigated the relevance and potential of linking environmental and health strategies and projects at the municipal level in Germany [9].

There have been several initiatives related to HHAPs in recent years. In 2020, the German health minister conference (the annual conference of all federal state health ministers to coordinate and agree upon health-related topics) passed a lead motion on climate change, with the aim of, among others, encouraging work on HHAPs at the municipal level [10]. The Lancet Countdown Policy Report for Germany 2021 highlighted the need to take action with regards to a legal anchoring of HHAPs, including a clear designation of the PHS as a key actor for the heat response [11]. This recommendation is in line with the proposal from the “federal ad-hoc working group on adaptation to the impacts of climate change in the health sector” that published eight core elements for the implementation of HHAPs in Germany in 2017, based on WHO recommendations. These eight core elements are (I) lead body and interdisciplinary cooperation, (II) use of heat alert system, (III) information and communication, (IV) reducing heat indoors, (V) particular care for vulnerable population groups, (VI) preparedness of the health and social care system, (VII) long-term urban planning and building sector and (VIII) monitoring and evaluation of measures. They include short-term action (such as specific responses to periods of heat waves), prevention during and before the summer, but also long-term action on development, planning and evaluation of HHAPs [12]. It also designates the PHS as the potential lead actor at the federal state level and as a possible coordinating institution for the implementation of heat measures at the municipal level. The lead actor would be responsible for initiating collaboration between all relevant stakeholders, comparable to existing disaster management structures [12]. These recommendations align with international developments, such as the International Association of National Public Health Institutes calling to strengthen the role of national public health institutes as key actors for climate change and health [13].

### The public health service in Germany

The public health service (PHS; *Öffentlicher Gesundheitsdienst*) is often referred to as the third pillar of the German health system, alongside inpatient and outpatient care [14]. Germany comprises 16 federal states and the work of the PHS in Germany is mostly defined by the scope of duties set out in the respective federal state law [14, 15].

The PHS in Germany includes agencies at national, federal state and municipal levels, with 377 municipal public health agencies (*Gesundheitsämter*). The PHS in Germany is publicly funded, in addition, it is possible for the PHS to charge fees for certain tasks [16]. There is no systematic assessment of the resources spent on the PHS, but it has been estimated that they account for less than 1% of overall health expenditure in Germany [14].

The main tasks of the PHS fall into the following three broad categories:

1. Health protection, including infectious disease prevention and environmental health;

2. Health promotion and disease prevention;

3. Health management, including tasks for the planning and improvement of the health system and quality assurance [14].

Over the past few years, the PHS has been facing several challenges, including an ageing workforce [14], insufficient digitalization [17] and a lack of resources and capacities, in particular for monitoring and coordinating activities [18].

Against the backdrop of the coronavirus disease 2019 (COVID-19) pandemic, these limitations of the PHS have been acknowledged and started to be recognized by political actors. A pact to strengthen the PHS (*Pakt für den ÖGD*) has been adopted by the federal and national health ministries, with the aim of preparing the PHS for new health crises in the coming years [19]. In the first report by the scientific advisory board accompanying this pact, climate change-related health consequences were identified as a key future health crisis [19]. However, none of the above-mentioned studies on HHAPs explicitly focus on the role or (self)perception of the PHS in Germany.

The aim of our research was to assess the current role of the PHS in the implementation of HHAPs at municipal and federal state levels in Germany. The specific objectives were:

1. To ascertain to what extent the work on HHAPs is included in the scope of duties of the PHS according to the legislation of the 16 federal states;

2. To analyse barriers and enablers within the PHS for a successful coordination of or collaboration in the implementation of HHAPs;

3. To determine whether actions targeted at vulnerable populations are prioritized by the PHS in the implementation of HHAPs;

## Methods

To assess the role of the PHS in the implementation of HHAPs in selected municipalities and federal states, a qualitative research design was used and comprised two distinct components: a policy document analysis of the legal frameworks for the work of the PHS in the 16 federal states, using content analysis, and semi-structured interviews with selected experts on their perceptions regarding the role of the PHS in the implementation of HHAPs in Germany, using reflexive thematic analysis.

Although the aim of this study was to assess the role of the PHS in the implementation of HHAPs, the role of the PHS in implementing singular or multiple heat prevention and response measures undertaken by the PHS were also explored, even if they were not implemented within the framework of HHAPs. These are referred to as “heat measures” in the following paragraphs. Importantly, we did not set out to document these in a comprehensive manner. As the definition and concept of the PHS can be ambiguous [20], in this study PHS is defined as the public agency or structure with a mandate for delivering public health services. For the German setting, this refers to the *Öffentlicher Gesundheitsdienst*. At the municipal level, the PHS operates through public health offices or agencies (*Gesundheitsämter*).

### Research setting

Data collection for this study was undertaken between May and July 2022 in Germany. The PHS in Germany includes agencies from the national, federal state and municipal levels. The 377 municipal public health agencies vary in size from less than 20 up to more than 100 employees [21]. According to the respective legal framework, the precise roles and duties of the PHS vary between the 16 federal states. 

### Policy docment analysis

Following an initial literature review regarding the role of PHS in HHAP implementation in Europe, the legal frameworks for the work of the PHS in the 16 federal states of Germany, that is, the health service laws (*Gesundheitsdienstgesetze*) or similar binding regulations, were sourced (see Additional file 1 for details). We decided to focus on health service laws, as these are currently the main reference for the scope of work for the PHS in the corresponding federal state, with few exceptions, such as infection prevention and control or drinking water guidelines.

In June 2022, these legal frameworks were retrieved from the individual websites of each federal state. These were then screened to identify whether and to what extent the following topics were mentioned: health and environment in general, climate and/or heat measures, disaster management or civil protection (hereafter referred to as “disaster management”). The aspect of disaster management was included because of the discussions around the heat response and its interconnection with emergency services and disaster management in Germany [11]. For the analysis, the legal frameworks were screened for any mention of paragraphs related to the above-described topics. Any relevant paragraphs mentioning any of the terms were extracted, categorized and compared with regard to differences in scope compared with the other regulations by one author (K.G.) and checked for consistency by a second author (S.V.). The method of analysis was content analysis as described by Hsieh and Shannon [22], using inductive category development. The detailed coding rules can be found in Additional file 2.

### Semi-structured expert interviews

To identify barriers and facilitators for the PHS to be involved in the implementation of HHAPs in Germany, expert interviews were conducted.

#### Identification, selection and recruitment of experts

The participating experts were identified on the basis of purposive sampling, focusing on representation across different levels of administration (that is, national, federal state and municipal level) and covering different perspectives [that is, PHS, other public institutions concerned with HHAPs, academia and non-governmental organizations (NGOs)]. Additional criteria considered during the selection process were variation in geographical location (that is, distribution of locations across Germany, and representation of urban and rural settings) and in levels of responsibility (that is, technical experts and managers). Participants were excluded if they did not have any experience with heat measures and/or working for or with the PHS.

Some experts represented two or more levels (for example, a representative from a national professional association who worked in a municipality) or also different sectors (for example, an expert worked in the PHS, but is also affiliated with a university). While several experts were identified because they were active in a flagship project on heat measures, experts from settings with relatively limited action to date (according to their own judgment) were also included. Given the small number of individuals working in this field, only limited personal information is displayed, and quotes are not assigned to specific roles to ensure the anonymity of participants.

For recruitment, experts were contacted via email. If there was no response, the experts were reminded of the request once and otherwise replaced by experts with a similar profile. We did not explicitly strive for data saturation; instead, the number of experts was chosen on the basis of pragmatic considerations [23].

#### Piloting and adaptation of the interview guide for semi-structured interviews

A guide for semi-structured interviews was developed on the basis of barriers and facilitators identified in the implementation of HHAPs in a previous study [8], complemented by questions addressing the specific role of the PHS according to the German national recommendations [12] (see Additional file 2). The interview guide was piloted with one expert and minimally adapted on the basis of the feedback received. The test interview was included in the analysis. As the level of responsibility of the interviewees differed, the guide was used flexibly to fit with the position and experience of the respective expert.

#### Conducting the expert interviews and transcription

Experts who had agreed to participate were invited for an interview in June–July 2022. The interviews were conducted in German by the lead author (K.G.). Following informed consent of the interviewees, the interviews were recorded using the videoconferencing software (Zoom) or a similar recording device in the case of a telephone interview or videoconferencing tool without a recording option (Webex). All participants agreed to the recording of the interviews. In addition, the interviewer took notes in the form of a memory protocol. The interviews were transcribed by an audio transcription service [24] and pseudonymized (with regards to real persons and organizations). All transcripts were double-checked by the lead author with the audio record and sent to the interviewee to give them the opportunity for validation (member check) and for possible additions or corrections of the transcript.

#### Qualitative analysis of the expert interviews

The transcripts were analysed using reflexive thematic analysis (TA) as outlined by Braun and Clarke [25]. This method was chosen, as it is considered to be a straightforward approach for exploratory analysis by recognizing themes and identifying patterns of meaning within the collected data [26]. The six distinct phases of thematic analysis (TA) include: (1) reading and re-reading the interviews to become familiar with the data, (2) generating initial codes, (3) merging codes into overarching themes, (4) Checking whether the overarching themes depict the data, (5) defining and describing each theme, and (6) producing the final report [25]. This was done by the lead author with regular input from the whole research team, for example, on the coding frame, the initial themes and possible conflicting topics, also allowing for overall reflections on the process. For steps 1–3, Microsoft Word was used [27]; for steps 4–6 the transcripts were imported into the qualitative data analysis software MAXQDA [28]. Before analysis of the data, on the basis of the recommendations given by Braun and Clarke [25], the following choices were made: The aim of the present study was to provide a detailed description of the data collected regarding the role of the PHS in the implementation of HHAPs in Germany, rather than describing all elements and aspects that came up in the interviews. A theoretical TA approach was chosen, as the lead author had some preconceptions and assumptions about possible barriers and facilitators that influenced the analysis. A semantic approach was applied to identify the explicit meaning of the data, in accordance with an essentialist paradigm, which is useful for a straightforward theorizing of findings from the data. Throughout the research process, the reflexivity of the lead author as the researcher in relation to the study topic was considered and documented. This included, among others, observations on recruitment process, data collection and analysis. This process is relevant, as a researcher in qualitative research is closely involved in the research process and might influence the aforementioned research steps [29]. During the conduct and analysis of the interviews in summer 2022, Germany experienced a new record in high temperatures. Therefore, it was necessary to postpone several interviews because it was either too hot for interviewees or because some interviewees were too busy because of urgent action related to the heat wave. In parallel, there was a lot of media attention on the topic. Most likely, these circumstances have influenced the perceptions on the urgency of the topic. To ensure accuracy and validity of the data, the means of establishing trustworthiness in reflexive TA as described by Nowell et al. were applied, including thorough documentation of code development, reflexive journal, informal peer debriefing and researcher triangulation throughout the phases of reflexive TA [30].

Reporting is based on the Standards for Reporting Qualitative Research (COREQ) [31]. The research team is an interdisciplinary team with a background in medicine (K.G.), public health (K.G., S.V. and E.R.), epidemiology (E.R.) and social sciences (S.V. and B.R.). All authors have experience in conducting qualitative research projects.

### Ethical considerations

Since the expert interviews contained exclusively technical questions, study-related stresses and risks were expected to be minimal. As some information provided by participants might involve criticism of their own agency or partner organizations, the pseudonymity of data was ensured, and no detailed information was disclosed. The study was conducted in compliance with the ethical principles for medical research involving human subjects set out in the Declaration of Helsinki [32]. It received ethical approval from the ethical committees of the London School of Hygiene & Tropical Medicine (LSHTM; reference number: 26796) and the Ludwig-Maximilians-Universität München (LMU Munich; project number: 22–0184).

## Results

### Policy document analysis

For the policy document analysis, 15 federal state laws and one ordinance for the PHS were identified. An ordinance is a binding legal act that needs to be implemented. Unlike laws, an ordinance has not been agreed upon through a legislative process by the parliament but was adopted by the executive [33]. In the following, all legal texts, including the federal state laws and the ordinance, are referred to as “regulations”.

None of the regulations for the PHS in the federal states of Germany explicitly mentioned “heat” or heat-related tasks. Only one regulation, from Hesse, mentioned the effects of “climate” on human health.

However, all regulations covered environmental topics at least to some extent (Table 1): All regulations covered environmental topics in sections related to health protection. Less often, they were mentioned under tasks related to health promotion and disease prevention (8/16) or health reporting (5/16). Paragraphs under health reporting described the observation and evaluation of health conditions including influences from the environment on health. Sections under health protection covered a broad range of activities, from rather passive “observe and analyse”, “advice and inform population and agencies” to “stimulate measures for protection” and precise measures such as statements for new planning initiatives, quality assurance and site visits. It is notable that none of the formulations indicated concrete measures, but remained suggestive. The wording between the two categories of health protection and health reporting was sometimes similar; for example, “observe and analyse” as well as “develop adaptation measures “ were found under both categories. Activities in relation to health promotion and environment were often mentioned in cooperation with other actors (NGOs or other sectors) and mainly included population-based approaches that worked towards health promotion and preserving social and environmental conditions. It was not specified whether these environmental conditions refer to natural or human-made elements. The paragraphs addressing tasks in cooperation with other institutions covered different aspects, such as the task of defining limits/benchmarks for certain harmful agents by state actors outside the health sector (for example, environmental agencies) and explicitly calling for cooperation of health agencies with other non-health actors on environmental topics in general. Overall, only few regulations indicated which part of the PHS, the municipal or the federal state level, was responsible for the actions; instead, most of the regulations just referred to “the public health service”.

**Table 1:** Federal state regulations and coverage of topics related to environment and health and disaster management

	Tasks related to environment and health				Tasks related to disaster management	
Federal state	Health reporting	Health protection	Health promotion and disease prevention	Tasks in cooperation with other institutions	Disaster management in general	Surveillance of hygiene of disaster management activities
Baden-Württemberg		x			x	x
Bavaria	x	x	x	x		
Berlin	x	x			x	
Brandenburg		x		x		x
Bremen	x	x	x	x		
Hamburg		x	x	x		x
Hesse		x			x	
Lower Saxony		x				
Mecklenburg-Vorpommern		x	x	x		x
North Rhine-Westphalia		x	x	x		
Rhineland-Palatinate		x		x		x
Saarland	x	x	x			
Saxony		x	x			x
Saxony-Anhalt		x		x	x	
Schleswig–Holstein	x	x	x			
Thuringia		x				x

The regulations cover disaster management to varying extents. Three regulations explicitly mention disaster management as a task for the PHS, six state the hygienic surveillance of disaster management activities and one covers the contribution of the PHS to the coordination with other actors involved in disaster management. In contrast, six regulations do not mention disaster management at all.

### Expert interviews

Overall, 12 interviews were conducted between June and July 2022 with 16 experts from different geographic locations (northern, southern, eastern and central Germany), at different levels (national, federal state and local), and with different sectors (PHS, environmental agencies, research, NGOs and professional association). One person approached for an interview sent feedback via email, which was not used for analysis, as it contained very limited information, and one organization did not reply; both were replaced with other persons from a similar institutional level or from the same geographic area. The interviews lasted between 34 and 67 min.

All interviewees reported to have relevant experience with the implementation of heat measures and that they were a key informant on the topic within their institution. The level of experience ranged from theoretical experience through self-study and/or online trainings and workshops to extensive practical experience with the planning and implementation of heat measures.

The interviews covered a broad variety of topics related to PHS and HHAPs in Germany, which are outlined in a thematic map (Additional file 4). In the following section, the three main themes “role of the PHS”, “targeting vulnerable populations” and “barriers and facilitators for the PHS” and respective codes are presented. These were repeatedly mentioned during the interviews, and considered to be of primary interest in answering the primary research objective. The focus of the interviewees’ response was on the municipal level, as this level is responsible for implementing HHAPs.

### The role of the PHS in the implementation of HHAPs

The first main theme was the description of the role of the PHS in the implementation of HHAPs or heat measures. Most interviewees stated that this role varies and that there is not one specific role for the PHS. As possible reasons for these variations, geographic location, capacities and resources, as well as local needs and requirements were named.

It is also handled differently. Depending on how the resources are in the health offices/So, if you now start from the municipal health offices. How the resources are. How the health offices are set up. Whether it is more in urban or rural regions. There is a wide range […] (P2).

Interviewees often differentiated between the role the PHS should ideally take (the “ideal role”) and the role the PHS is currently taking (the “real role”).

Regarding the real role, it was commonly noted that the PHS at the municipal level did not initiate or coordinate HHAP activities but rather supported and contributed to projects that have been initiated by other actors (as a “consultant”). Often, it was stated that the initiating municipal actors were environmental agencies. An inherent ownership for climate change related topics, experience from flood management, more structural support from the federal state and national level agencies, better funding schemes and greater success in applying for these were mentioned as possible reasons for why environmental agencies often take the core role. One interviewee stated:

Because the topic of climate protection is mostly in their [environmental agency] domain … where climate protection has traditionally been located. … And my experience is that in some cases they see it quite naturally or organically as their task to also take care of climate adaptation. And in fact, climate adaptation, i.e. flood prevention and all these plans that already exist, just as an example, is actually also an environmental, i.e. a classic task for the environmental agency… climate adaptation is already perceived as, yes, a task of the environmental side, and that’s why they also devote themselves to heat protection. (P8).

Underlying this general observation, some participants even stated that the municipal PHS would not be able to take on a coordinating or lead role for HHAPs. When asked under which circumstances this would be possible, one interviewee explained:

This [the HHAP] has a dimension because it is [federal state]-wide, which is not my responsibility, or not the responsibility of the PHS. So the PHS/I rather make sure that we are involved everywhere when something is going on, that we are not forgotten again, as it is happening right now, but in the end I can only try to be heard at my [municipal] level, and perhaps form an internal climate working group here. (P13).

For the federal state level, interviewees reported that their main tasks were providing advice, informing and connecting different stakeholders, either at the federal level or at both federal and municipal levels. One federal state health agency reported to be leading initiatives with regards to heat measures. However, it also became evident that the federal state level could only support the municipal level in the implementation, for example, through the provision of information, and that other opportunities for practical implementation were very limited.

The role of the PHS at the municipal level was described in differing ways by the interviewees: Several times the PHS was mentioned as an implementor of communication measures. There were different explanations of what these might look like; for example, one participant stated:

One of the core elements of all heat action plans is basically to raise awareness among the population, and […] basically it is primarily about behavioural prevention. And in this health education, the PHS can fulfil its task very easily and very low-threshold with few resources. (P4)

In contrast, another interviewee said:

What many municipalities do is: “Well, we update, we have handouts on our website”. That just doesn’t help. So, it’s good that they exist and flyers are important, but not in the way they are normally provided, namely just quietly somewhere on some sub-page, in the very margins of the municipal website. That is not an appropriate way of communicating and warning the population. (P8).

A third participant from the municipal PHS explained their work and the relevance of the PHS as follows:

So, I wanted to say again that the public health service has an important function because it looks at the public and the individual target groups and develops measures that go to the people, so to speak, and can often design short-term or medium-term measures there, and it simply has to be involved. So, it must also be considered in the concepts, because WE have the contact to the stakeholder level, and we have the contact to the city districts […]. (P13).

Overall, there was consensus among all interviewees that the PHS should be involved in the implementation of HHAPs. The three main reasons for this mentioned by the interviewees were the key role of the PHS in connecting health stakeholders with public institutions; its strong existing connections and networks with other multipliers and vulnerable population groups on the municipal level; and that, overall, the PHS could be a strong actor to implement a Health in All Policies (HiAP) approach (which was mentioned with reference to the “ideal role” of the PHS):

I also see a very important role for [the PHS] in the future in the coordination of preventive measures, but also of measures in acute situations, which we always have. […] And then, of course, I see it [the PHS] ideally under the aspect of health in all policies in every urban planning measure. (P10).

The PHS was also seen as the ideal actor for controlling quality of implementation of the HHAPs, for example, by checking on the implementation of heat measures in nursing homes or hospitals.

### Taking into account the needs of vulnerable populations

There was a broad consensus among interviewees that vulnerable populations would need to be the primary beneficiary group of heat measures. Several interviewees explained that in this context the word “vulnerable” primarily referred to those groups that are not as capable of helping themselves and who suffer most from heat effects. When asked about their characteristics, there was an agreement among interviewees that these groups included in particular older and chronically ill people, but also pregnant women, small children and those in socially disadvantaged situations, such as homeless people.

#### Dimensions affecting PHS engagement with the implementation of HHAPs

**Fig. 1:**
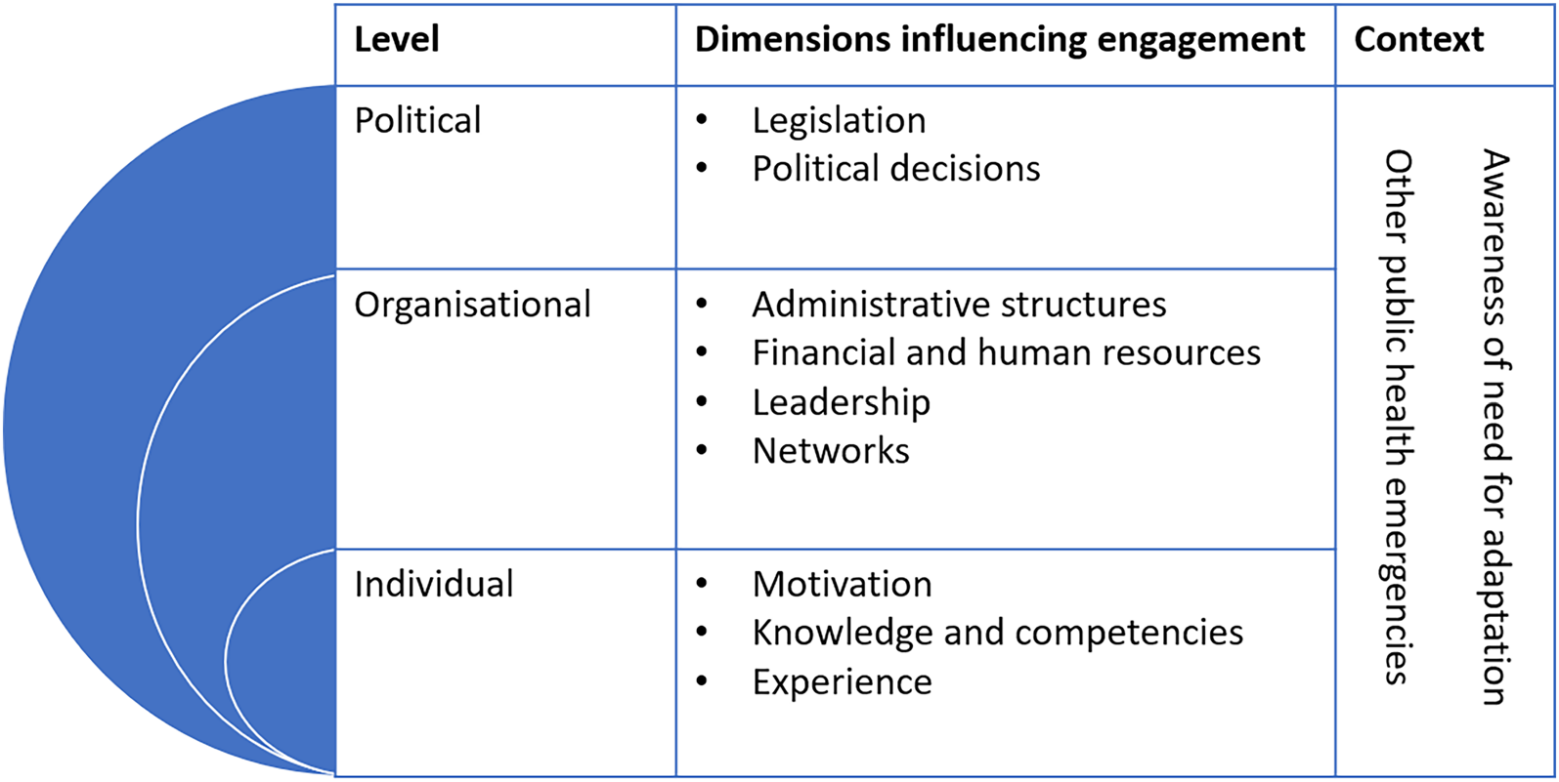
Dimensions affecting engagement of the public health service (PHS) with the implementation of heat health action plans in Germany at political, organizational and individual levels. Dimensions (absent or present or operating on a spectrum) may act as barriers to or enablers of PHS engagement with implementation. For simplicity, these dimensions are assigned to one level but some affect multiple levels (for example, administrative structures)

The largest theme covered in the interviews were barriers and facilitators in relation to the implementation of HHAPs by the PHS. While we had set out to distinguish between dimensions influencing the PHS role in a positive or negative manner, this turned out not to be feasible: All dimensions operate on a spectrum and may thus act as both a barrier to or an enabler of PHS engagement with HHAP implementation. This theme was examined at three main levels, at the individual level (the person working on the implementation), the organizational level (the PHS as an authority) and the political level (the political discourse at the municipal/federal state level). The three levels are interconnected in multiple ways and some of the identified codes fit into more than one. Two contextual elements, awareness of the need for climate adaptation and other public health emergencies, were identified in the interviews that influenced the implementation across different levels (Fig. 1).

One contextual factor was the presence of other “public health emergencies” such as COVID-19, monkeypox or the high number of refugees from Ukraine, which were described as a major barrier for the PHS in prioritizing non-mandatory tasks, such as HHAP implementation, on the individual, organizational and political level.

In addition, a major facilitator that was mentioned across the different levels was awareness of the need for adaptation: persons and organizations, but also political parties “who understood the importance of the topic” (P8) and what they could do to address it, were taking action on heat measures or supporting the PHS in taking an active role.

And in this way, decision-makers in municipal administrations can also take action and accept this as a problem or a challenge, or as those who say: “I take responsibility for implementing heat protection in this area.” (P8).

At the individual level, closely linked to awareness, motivation to get involved was mentioned as an important factor that could either enable action if present or hamper implementation if absent. Knowledge on climate change and health topics generally, and on heat measures specifically, and competencies to implement certain measures of HHAPs, for example, organizing and coordinating stakeholder action, were also identified as relevant factors.

In very few health offices there are people who have already dealt with this topic [heat and health] more intensively. And if I want to implement something like this, then I must either have someone who can do it or at least someone who is willing to qualify in order to be able to implement it. And in this respect, I see a big, big point in the qualification of the existing staff. (P14).

In municipalities that were considered to be good practice examples, these competencies and knowledge were either already present among the staff because of previous work, or it was possible to hire a person with this specific skill set. It was also noted that in municipalities without HHAP-specific knowledge and competencies in the PHS cooperation with other agencies could be a solution to overcome this gap, as expressed by one participant:

We alone do not have the entire expertise. So, in my opinion, it only works in a network, only in cooperation. (P11).

Interview participants highlighted the availability of information materials, workshops and exchange opportunities provided by NGOs, universities, public agencies and other training institutions in Germany as a valuable resource for further individual education and training. It was reported in some interviews that individuals independently educated themselves on the topic of climate change and health. However, it was also critically noted that there is a need to include these topics in mandatory curricula for professionals working in the PHS:

And I would really like to see more input from the [training institutions for PHS professionals]. […] I found what was going on in the area of environmental medicine very disappointing. It was a day on sick-building syndrome and MCS [Multiple chemical sensitivity], and it was somehow like that. And I would have liked to see a lot more input and awareness-raising. [...] And I believe that the future medical public health officers [Amtsärzte] should also be trained accordingly. (P11).

At the organizational level, defined here as the public health office and the overarching administrative structure, a number of barriers and facilitators were identified. One major barrier often mentioned was related to the rigid, non-agile administrative structures, which hindered work between agencies and with external actors, leading to frustration at the individual level, less cooperation at the organizational level, and working in silos at the political level. One interviewee described this as follows:

The problem with interagency cooperation is always the area of competence and the degree of effectiveness, and if the PHS or the coordinating body, which can also be the environmental agency, is not granted this competence, then the degree of effectiveness is very low. (P4).

Financial and human resources were the two closely interlinked elements. Where available in sufficient quantity and quality, they were identified as a facilitator for success, and where absent, as a reason for hampered implementation. The difficult situation of the PHS with regards to staff capacities was often mentioned, in line with the high workload due to other public health emergencies. Municipalities that were considered good practice examples were able to respond to a shortage in internal funding by successfully sourcing external funding either for material or for additional staff positions. Often the PHS did not have the leading role in applying for external funding but was supporting or advising applications by environmental agencies. Interviewees revealed several reasons for this, ranging from the limited knowledge in the PHS about funding schemes, limited capacities and resources to apply for funding and sometimes rigid funding schemes. One interviewee acknowledged this challenge and explained that this was not only applicable to funding for heat measures, but in general:

We are now seeing this with the funds that are being made available to promote the public health service. So, a group that has been busy to the hilt lately, so to speak, to sit in front of a pot of money and say, now write applications within, yes, a few weeks about the things that you have been lacking for years. (P3).

Previous positive experiences in applying for external funding, as well as persons working on project positions funded by third parties, were mentioned as possible facilitators for obtaining external funding. Another facilitator in this regard was the use of networks with actors from outside the public health office, for example, through collaborations with local multipliers, universities or NGOs or through public–private partnerships to overcome the lack of capacities and resources.

The interviewees mentioned that several organizational aspects depend on the location of the respective agency, as the geographic setting could make a difference in terms of vulnerability to heat events. Likewise, the size of the municipality, as well as the size of the public health office, determines financial and human resource capacities. Regarding the difference between urban and rural settings, several participants noted that the PHS in rural areas had limitations with regards to staff and financial resources and, consequently, knowledge and competencies. On the other hand, it was explained that the smaller size of the public health office could make it easier to connect with other agencies and to implement certain measures; joining forces with other municipalities was suggested to be a good opportunity.

Leadership and support by supervisors were often mentioned as driving forces for the implementation of heat measures. While not all interviewees from municipalities reported an office-wide supportive environment to implement measures, it was often reported that the direct supervisors supported and encouraged such work. In addition, there were also comments regarding the necessity of supportive managerial structures in general, such as the following:

And then there are public health offices that have a very flat hierarchy and office management or departmental management, unit management, which allow their staff a lot of leeway and also room for manoeuvre and also room for growth and competence acquisition. (P4).

This aspect was not explicitly expressed during the interviews by all participants, but intriguingly, strict hierarchical structures were mentioned as a common barrier during informal conversations with the lead researcher after two of the interviews.

At the political level, macro-level determinants influencing the implementation of heat measures were mentioned. One major element was the legal foundation and the policies for the PHS in the respective federal state. While it was acknowledged that the legal foundation is important for HHAP implementation, there was no consensus among interviewees on whether the current legislative basis was sufficient or not. For some interviewees, it was evident that a strong legal framework that explicitly mentions heat measures and that clearly assigns roles and tasks, would be necessary to enable action by the PHS.

Because I think that’s what it [the law] is for, again, especially with regard to surveillance systems, for example, or the whole monitoring process. Which would also be a cornerstone for heat protection measures. But also to be able to react quickly in an emergency. The whole thing is not represented in/by law. (P6).

In contrast, other participants stated that the current legislation provides the opportunity for the PHS to become engaged with the implementation of HHAPs and that, even with a stronger legal framework, the implementation would not necessarily improve.

Well, it’s super easy to cry out for the legislator and say, yes, he has to make a law now and then everything will be okay again. Because the question is, what do you want to write into this law? […] I think, as I experience it, my impression is that everybody there would like to contribute. And it is much more important to have the manpower to inform and to network. And I think a lot would happen then. I don’t know if so much needs to be regulated by law. (P3).

This was supported by the statement of another interviewee, who criticized a top-down approach:

And I have the feeling that these are issues that have to be dealt with now because there is a legal regulation and someone had to take over. And this is now, these are not people who are specifically responsible for climate. They got it on top of that a little bit.” (P13).

However, it was argued that legislative accountability would lead to more financial and human resources:

It would be easier if this duty existed, then there would be resources, sufficient human and financial resources, and it would be given a completely different priority in the budgets, if it was part of the compulsory tasks. (P8).

Aside from the legal frameworks, interviewees also mentioned political support in terms of decisions and processes as relevant factors for driving the implementation of heat measures. Some participants explained that the initiative for the project came from the upper-level health ministry; others elaborated that they involved the city council with the development of the plan:

We had always included political actors in the process. Right from the start. […] However, these actors were involved in the overall process, so that they could also include opinions, impulses and expert opinions, because of course they also ultimately decide on this and the concept. And our decision went through without a veto. (P7).

## Discussion

The policy document analysis examined the role of the PHS in the implementation of HHAPs in Germany, considering heat measures as a responsibility at the interface between environment and health issues and disaster management. The analysis revealed that the word “heat” was not included in any of Germany’s federal state regulations, while the term “climate” was only present in 1 (Hesse) out of 16 federal state regulations. PHS responsibilities in the field of environment and health were described in all federal state regulations, but with varying scopes of duties and – according to the expert interviews – without any requirement for implementation. These findings suggest that there is currently no accountability mechanism in place to enforce involvement of the PHS with the implementation of HHAPs. In addition, the regulatory basis does not clarify which role the PHS should take in the implementation of HHAPs, and a clear definition of tasks is lacking.

The analysis of the responsibility of the PHS in disaster management presents a rather scattered picture, with very different levels of engagement prescribed by the regulations in different federal states, ranging from not being mentioned at all (in six regulations) to a broad involvement of the PHS in disaster management (in three regulations). These findings suggest that currently there is only a weak legal basis for the PHS to take an active role in disaster management. However, the emergency aspects of the heat response should not be omitted, and the interlinkages between the areas of disaster management and preventive measures could be strengthened. Researchers from Melbourne, Australia, found a tension between the conceptualization of heat as an emergency versus understanding heat as a source of chronic stress, but also concluded that it is inevitable to bring these two fields together, thereby improving the heat response overall, with special consideration of social vulnerability [34].

The lack of accountability and clarity regarding the role of the PHS identified in the policy document analysis helps explain the findings from the interviews. The interviewees noted that the PHS does not have a clearly specified role, and that its actual scope of work in this area often depends on local circumstances. Interviewees also pointed out that it is currently not the PHS, but the (local) environmental agencies that tend to take the lead on municipal activities related to heat measures. It also became evident that it is important to include the PHS as an actor in the planning and implementation of HHAPs and that there is much potential in empowering the PHS to take a more active role. The challenges described by participants confirm the quantitative findings from previous studies on HHAP implementation in municipalities, where lack of financial and personnel resources were rated as main barriers for the implementation, followed by lack of political will and lack of concern [8]. Similarly, a publication assessing the status of environment and health strategies at German municipal levels identified political support and organizational leadership as facilitators, as well as a culture of cooperation and communication within the municipality but also with external stakeholders [9]. Despite being published in 2005, these factors were confirmed by our study as very relevant for the local implementation process.

The findings of this study are also in agreement with studies from other European countries: In an article by Vanderplanken et al., national/regional PHS agencies were only identified in 8 out of 15 countries as key stakeholders that need to be included for HHAP implementation; for Germany the PHS was not listed [35]. Casanueva et al. analysed heat-health warning systems in Europe and highlighted the PHS in general or national public health institutes in particular as relevant target groups, for example, in France, Hungary, Italy and the Netherlands [36], while for Germany the “health system” was mentioned in a general manner. A comparative assessment showed that the engagement of public health agencies in 22 large cities globally is often limited to heat warning and risk communication [37]. These findings suggest that public health agencies, including the PHS, are considered relevant actors; however, a role for them in HHAP implementation beyond the communication of heat warnings has only been described in selected countries. One reason for this lack of clear role assignment may be the fact that the structures and mandates of public health agencies vary considerably between and also within countries [20].

One of the major contextual factors for the PHS to take an active role mentioned across all levels was “awareness”, which covered aspects related to consciousness about the impact of climate change on health but also knowledge about possibilities to take action. This finding concords with the foundations for decision-making described by Jones and colleagues, who proposed that, in the early stages of response development for climate change in a community, the main focus should be on raising awareness to support decision-making, while later the focus should be on assessing conflicting goals in decision-making [38]. The “other public health emergencies” that were mentioned as a relevant contextual factor leading to a de-prioritization of non-mandatory tasks are closely connected to existing challenges of the PHS in Germany. The effects of the COVID-19 pandemic on the PHS have been described in other studies as exacerbating existing problems, such as the shortage of staff capacities, leading to the inability to fulfil mandatory duties [17]. The barrier “rigid and non-agile administrative structures”, which hampers successful bottom-up as well as top-down approaches, was pointed out previously by Austin et al. with regards to climate change adaptation and the PHS in Germany [39]. Even before the pandemic, limited human resources for tasks related to coordination and cooperation with other stakeholders were present in the PHS [40], and the results of the interviews confirmed that this also applies for tasks in the area of heat measures.

The results from the interviews confirm that the focus is on the needs of vulnerable groups. Whether these aspirations follow a proactive approach as recommended in the literature [5, 41] or are more reactive, as indicated by one of the interviewees with regard to communication materials, should be further investigated.

The findings of this study are also of relevance for climate adaptation measures and PHS beyond heat measures. A 2019 study by Woodhall et al. on public health adaptation to climate change in local municipalities in South West England identified a lack of remit for the PHS, limitations in resources and financial constraints as well as lack of leadership and awareness as main barriers to greater engagement of the PHS [42]. Findings from the USA also identified available funding, prioritization by the state or institution, the ability and capacity of staff and political will as determinants for the success of climate change and health activities undertaken by state health agencies [43]. In agreement with the findings of this study, several solutions have been proposed which can be broadly categorized into funding (for example, securing of adequate funding), knowledge and skills (for example, development of practical guidance, training of staff), organization (for example, implement multi-sectorial exchanges) and prioritization (for example, providing leadership on climate change and health within but also outside of public health agencies) [43, 44].

### Strengths and limitations

To our knowledge, this is the first study to analyse the role of the municipal and federal PHS in Germany with regards to heat measures, as well as to assess barriers and facilitators for engagement of the PHS in the implementation of HHAPs. A core strength of this study is its two-component approach, integrating an analysis of the legislative basis for the PHS role in this field, and findings from expert interviews. Each of these two components has its own strengths and limitations.

The policy document analysis comprehensively reviewed regulations for all of the 16 federal states. However, it did not analyse a broader range of policy documents, such as climate change adaptation regulations, which may discuss the assignment of roles in the context of heat measures. Also, it did not conduct an in-depth qualitative or legal analysis of the description of the tasks related to environment and health or disaster management. The results are nevertheless useful for providing an overview on the legal foundation of the PHS for tasks in the context of environment and health.

The expert interviews conducted at different administrative levels and institutions yielded a broad range of perspectives and important insights into barriers and facilitators for the involvement of the PHS in heat measures. The first author is actively involved in several networks seeking to strengthen public health and to develop a more interdisciplinary PHS in Germany. These networks and experiences helped with recruiting experts but might have also led to a selection bias. The broad sampling approach, which sought to include different institutions and administrative levels, ensured a multi-perspective sample but did not allow for the identification of any connected relations (for example, the presence of an explicit law on HHAPs/environment and health topics in one federal state and its implications for the implementation of HHAPs at the municipal level). Furthermore, the number of experts interviewed was based on pragmatic considerations [23], and it is conceivable that the conduct of further interviews may have yielded additional insights.

Thematic analysis allows for a broad range of analytical options and is therefore of advantage in the context of exploring new content and topics. This flexibility can also be challenging, as there is no clear indication for researchers on what aspect or level to focus on [25]. This challenge was addressed by clearly stating the underlying assumptions and the level of analysis before embarking on the data analysis. A further limitation was the absence of a co-coding person, which is not required for reflexive TA, but is generally considered to increase the rigour of the analysis in qualitative research [29].

## Conclusion

Sub-national health actors such as the PHS are relevant stakeholders for the implementation of measures to mitigate the effects of climate change on health, for example, through HHAPs. This study investigated the role of the PHS in the implementation of HHAPs in Germany. The results show that the legal framework for the PHS in the 16 federal states for activities with regards to environment and health or disaster management only provides a rough orientation and no firm basis for action. This is also reflected in differing roles of the PHS described by participating experts. Key facilitators that were identified were political support, adequate financial and human resources, supportive leadership, existing networks within the public agency and also with external actors, motivation and knowledge and competencies of individuals. These facilitators may support the PHS in strengthening its active role in the implementation of HHAPs in Germany and should be taken into consideration when planning the scale-up of HHAPs at the local level.

**Relevant translations**

**Table:** 

English	German
District office	*Landratsamt*
Health service law	*Gesundheitsdienstgesetz*
Public health service	*Öffentlicher Gesundheitsdienst*
Public health office	*Gesundheitsamt*

## Supplementary Information

Additional file 1.

Additional file 2.

Additional file 3.

Additional file 4.

## Data Availability

The dataset for the expert interviews generated and analysed during the current study is not publicly available to ensure the anonymity of study participants. Upon reasonable request, aggregated data without any personal information can be obtained from the first author.

## Abbreviations

HiAP: Health in all policies
HHAPs: Heat health action plans
HHWS: Heat-health warning systems
NGO: Non-governmental organizations
PHS: Public health service
TA: Thematic analysis
