# Supplementary material for: The role of the public health service in the implementation of heat health action plans for climate change adaptation in Germany: A qualitative study
Source: Health Res Policy Syst. 2024 Dec 5;22:161. doi: 10.1186/s12961-024-01231-6 (PMC11619655; doi:10.1186/s12961-024-01231-6)
Supplement: Supplementary file 1 — Additional file 1. [file 12961_2024_1231_MOESM1_ESM.docx]

# Additional file I websites for policy analysis

The following websites were used to extract the documents for the policy analysis in May 2022:

| **Federal state** | **Link** |
| --- | --- |
| Baden-Württemberg | https://www.landesrecht-bw.de/bsbw/search |
| Bavaria | <https://www.gesetze-bayern.de/Content/Document/BayGDVG-15> |
| Berlin | <https://gesetze.berlin.de/bsbe/document/jlr-ÖGesDGBErahmen> |
| Brandenburg | <https://bravors.brandenburg.de/gesetze/bbggdg> |
| Bremen | <https://www.transparenz.bremen.de/metainformationen/gesetz-ueber-den-oeffentlichen-gesundheitsdienst-im-lande-bremen-gesundheitsdienstgesetz-oegdg-vom-27-maerz-1995-72448?asl=bremen203_tpgesetz.c.55340.de&template=20_gp_ifg_meta_detail_d> |
| Hamburg | <https://www.landesrecht-hamburg.de/bsha/document/jlr-GesDGHAV8IVZ> |
| Hesse | <https://www.rv.hessenrecht.hessen.de/bshe/document/jlr-GesDGHEV4IVZ> |
| Lower Saxony | <https://www.nds-voris.de/jportal/?quelle=jlink&query=GesDG+ND&psml=bsvorisprod.psml&max=true&aiz=true> |
| Mecklenburg-Vorpommern | <https://www.landesrecht-mv.de/bsmv/document/jlr-%C3%96GDGMVpP29> |
| North Rhine-Westphalia | <https://recht.nrw.de/lmi/owa/br_text_anzeigen?v_id=10000000000000000042> |
| Rhineland-Palatinate | <https://landesrecht.rlp.de/bsrp/document/jlr-GesDGRPV4P5> |
| Saarland | <https://recht.saarland.de/bssl/document/jlr-GesDGSLrahmen> |
| Saxony | <https://www.revosax.sachsen.de/vorschrift/3348-SaechsGDG> |
| Saxony-Anhalt | <https://www.landesrecht.sachsen-anhalt.de/bsst/document/jlr-GesDGSTrahmen> |
| Schleswig-Holstein | <https://www.gesetze-rechtsprechung.sh.juris.de/jportal/?quelle=jlink&query=GesDG+SH&psml=bsshoprod.psml&max=true&aiz=true> |
| Thuringia | <https://landesrecht.thueringen.de/bsth/document/jlr-GesDVTH1998rahmen> |
