# Supplementary material for: The role of the public health service in the implementation of heat health action plans for climate change adaptation in Germany: A qualitative study
Source: Health Res Policy Syst. 2024 Dec 5;22:161. doi: 10.1186/s12961-024-01231-6 (PMC11619655; doi:10.1186/s12961-024-01231-6)
Supplement: Supplementary file 2 — Additional file 2. [file 12961_2024_1231_MOESM2_ESM.docx]

# Additional file II Coding rules

**Coding rules**

The four categories were developed inductively based on the findings of the data.

Health reporting

- Paragraphs that explicitly mention any term related to “environment” (“Umwelt”) in the context of “health reporting” (“Gesundheitsberichterstattung”).

Health protection

- Paragraphs that explicitly mention terms related to “environment” (“Umwelt”) and “protection” (“Schutz), “environmental medicine” (“Umweltmedizin”), “influences from the environment on health” (“Auswirkungen der Umwelt auf die Gesundheit“).

Health promotion

- Paragraphs that mention terms related to “health promotion” (“Gesundheitsförder*“), “prevention”, “tasks under social responsibility” (“Aufgaben in sozialer Verantwortung”) and “environment”,

Cooperation

- Paragraphs that explicitly mention cooperation with other non-health agencies/actors in the context of “environment” topics.
