# Supplementary material for: The role of the public health service in the implementation of heat health action plans for climate change adaptation in Germany: A qualitative study
Source: Health Res Policy Syst. 2024 Dec 5;22:161. doi: 10.1186/s12961-024-01231-6 (PMC11619655; doi:10.1186/s12961-024-01231-6)
Supplement: Supplementary file 3 — Additional file 3. [file 12961_2024_1231_MOESM3_ESM.docx]

# Additional file III Expert interview guide

| **General information** | | | | | | | |
| --- | --- | --- | --- | --- | --- | --- | --- |
| **Date** |  | **Start time** |  | **End time** |  | **Duration** |  |
| **Location/Software** |  |  |  |  |  |  |  |
| **Interruptions with time** |  |  |  |  |  |  |  |

| **Research management** | | |
| --- | --- | --- |
| **Investigator’s name** | | **Function** |
|  | |  |
| **Participant’s name** | **Function** | **Organisation** |
|  |  |  |

| **Interview instructions** | |
| --- | --- |
| - Check that the recording device is working and that the consent forms are available digitally.  - Welcome the interviewee and introduce yourself with your name and function.  - Thank the interviewee for taking the time to participate in the research project.  - Address the interviewee formally (“Sie” in German, rather than “Du”).  - Briefly explain the purpose of the research and the topic of the expert interview, as well as the process.  - Go over the consent form and have the interviewee sign it.  - Ask the interviewee if there are any questions and answer all questions before you begin.  - Collect the interviewee's personal information.  - Start the tape/online recording and then begin with the first question. | |
| **Research aim** | To assess the role of the Public Health Service in the implementation of HHAPs at the municipal and federal state level in Germany. |

*Please note that the order of the topics might change according to the flow of the conversation.*

| **No** | **Min** | **Open question** | **Possible deepening question** | **Prompt** | **Aim** |
| --- | --- | --- | --- | --- | --- |
| 0 | 3 | Please introduce yourself with your name and function, the level your work mainly focuses on (national, federal state, municipal) and your role with regards to heat health action plans (HHAPs) and/or the public health service in Germany. |  |  | Introduction & warm-up |
| 1 | 5 | What are your experiences with regards to the implementation of HHAPs in Germany? | In how far are HHAPs implemented in Germany at the different levels (i.e. national, federal state, municipality)?  Which federal state/municipality do you consider a good practice example that other parts from Germany can learn from?  What components are, from your perspective, easy to implement? Which ones are more challenging? | Why do you see it this way? | Experience with regards to the implementation of HHAPs |
| 2 | 10 | What role does the public health service play in the implementation of HHAPs in your municipality/federal state/area of expertise? | In how far does the public health service take a coordinating or leading role in the implementation of HHAPs in [the specific municipality/federal state]? What are other actors that could take over the coordinating role and why would these be better equipped for that?  From your perspective, how does the role change between the different components of the HHAPs?  What differences have you observed for the public health service on the different levels (national, federal state, municipal) with regards to HHAPs? | Do you have any practical examples to explain your impression? | Role of the public health service in the implementation of HHAPs (or individual HHAP components) on the different levels |
| 4 | 10 | What are specific barriers and enablers experienced by staff working in the public health service during the implementation of HHAPs on the municipal/state level? | How high is awareness for the relevancy of heat health adaptation and the responsibility of the public health system in the respective agencies? What are possible differences in the level of awareness depending on the level of hierarchy (e.g. within the agency) and/or level of responsibility (e.g. municipal, federal state, national)?  To what extent are people working in the public health service trained for taking over a coordinating role at the municipal/federal state level? What kind of competencies are needed for facilitating an exchange between all responsible actors, but also to show strong leadership?  Motivation of individuals has been shown to be a key driver for the implementation of measures in a previous study – in how far is this finding in line with your experience with the public health service? | Which of the factors you have mentioned would you see as most relevant?  Why do you see it this way?  Do you have any practical examples to explain your impression?  What could be practical solutions to the barriers you have just mentioned? | Barriers and enablers with regards to the public health staff |
| 5 | 10 | What are specific organisational barriers and enablers for the public health service to support the implementation of HHAPs on the municipal/state level? | In how far do financial (e.g. through third-party funding) or personnel capacities play a role?  To what extent do political decisions support and/or discourage the implementation?  Which legal frameworks support the implementation of HHAPs at the state/municipal level? | Which of the factors you have mentioned would you see as most relevant?  Why do you see it this way?  Do you have any practical examples to explain your impression?  What could be practical solutions to the barriers you have just mentioned? | Barriers and enablers with regards to organisational aspects |
| 6 | 5 | To what extent do the current implemented elements of HHAPs focus on the vulnerabilities of different populations within your municipality/state/area of expertise? | Which role should the public health service take, from your perspective, for prioritizing needs of vulnerable populations? | What are reasons for this?  What are practical solutions? | Prioritization of vulnerable populations for the implementation of HHAPs |
| 7 | 3 | Are there any additional aspects you would like to talk about with regards to the implementation of HHAPs and the public health service? |  |  | Closing Question |

.
