# Supplementary material for: The role of the public health service in the implementation of heat health action plans for climate change adaptation in Germany: A qualitative study
Source: Health Res Policy Syst. 2024 Dec 5;22:161. doi: 10.1186/s12961-024-01231-6 (PMC11619655; doi:10.1186/s12961-024-01231-6)
Supplement: Supplementary file 4 — Additional file 4. [file 12961_2024_1231_MOESM4_ESM.docx]

# Additional file IV Thematic map of the content covered during the interviews


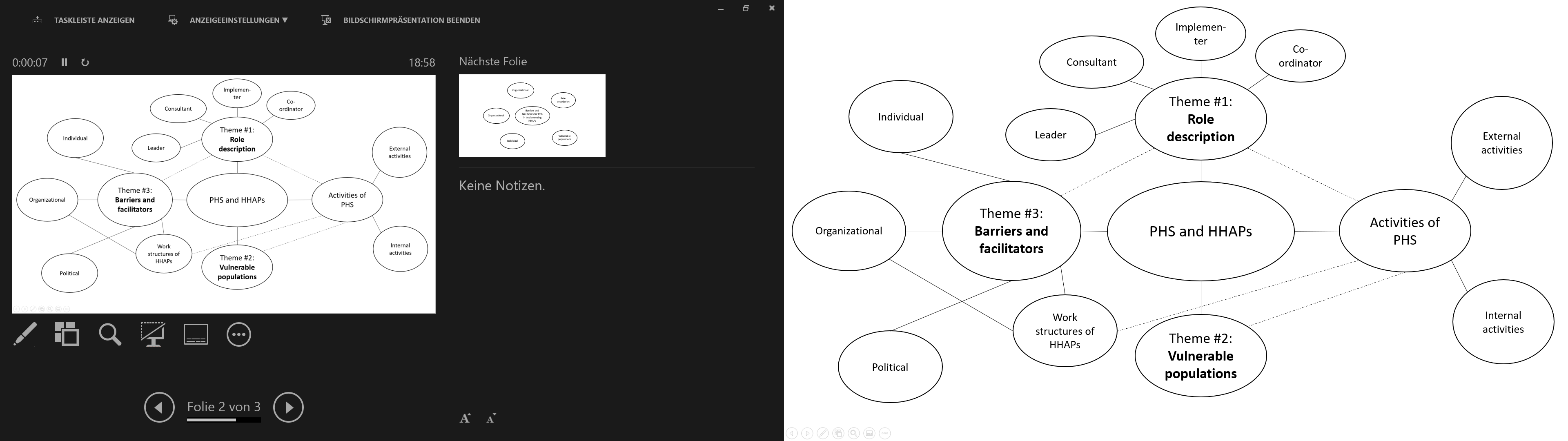


**Legend**: Themes highlighted in bold are further explored in the study; dotted lines show indirect connections, clear lines direct connections
